# Rapid Enlargement of Squamous Cell Carcinoma Arising From Hidradenitis Suppurativa During Bimekizumab Therapy

**DOI:** 10.7759/cureus.92172

**Published:** 2025-09-12

**Authors:** Yuto Yamamura, Kazuyasu Fujii, Chisa Nakashima, Shunya Usui, Kazutoshi Nishimura, Atsushi Otsuka

**Affiliations:** 1 Dermatology, Kindai University Hospital, Ōsakasayama, JPN

**Keywords:** bimekizumab, hidradenitis suppurativa complication, hidradenitis suppurativa (hs), interleukin-17 a inhibitor (il-17ai), squamous cell carcinoma (scc)

## Abstract

Hidradenitis suppurativa (HS) is a chronic inflammatory skin disease that can rarely transform into squamous cell carcinoma (SCC), often with aggressive clinical behavior. Biologic agents, including interleukin-17 (IL-17) inhibitors, are increasingly used for severe HS, but their effects on tumor surveillance remain unclear.

We report a man in his 50s with paraplegia and a 20-year history of Hurley stage III HS affecting the buttocks and perineum. Four months after initiating bimekizumab for uncontrolled HS, a chronic ulcerative lesion on the left trochanter rapidly enlarged into a 15 cm exophytic tumor. Biopsy confirmed SCC, and biologic therapy was discontinued. Wide local excision with 2 cm margins and sentinel lymph node biopsy were performed, revealing high-grade invasive SCC with negative margins and no nodal involvement. Reconstruction was achieved using a split-thickness skin graft.

This case illustrates the malignant potential of chronic HS, particularly in long-standing Hurley stage III disease, and raises concern for the possible acceleration of tumor growth during biologic therapy. Although large studies have not shown increased cancer risk with interleukin-17 inhibitors, vigilance is required when treating patients with HS with biologics. Rapidly progressive or non-healing lesions should prompt early biopsy to exclude malignancy. To our knowledge, this represents the first reported case of HS-associated SCC arising during bimekizumab treatment, emphasizing the importance of careful monitoring in this population.

## Introduction

Hidradenitis suppurativa (HS) is a chronic, relapsing inflammatory skin disease, often associated with risk factors such as smoking, obesity, and family history, that, though uncommon, can give rise to squamous cell carcinoma (SCC) after long disease duration [1]. HS-associated SCC typically demonstrates aggressive biologic behavior, with high rates of metastasis and disease-specific mortality [2].

Biologic therapies, including tumor necrosis factor-alpha (TNF-α) inhibitors (e.g., adalimumab) and interleukin-17 (IL-17) inhibitors (e.g., bimekizumab), have become standard treatments for severe HS [3]. However, their immune-modulating effects raise theoretical concerns about impaired tumor surveillance and the facilitation of malignancy in susceptible patients [4].

An increased risk of malignancy has been reported with TNF-α inhibitors [5], whereas IL-17 inhibitors are generally considered to carry a lower risk [6]. To date, no cases of HS-associated SCC exacerbated during IL-17 inhibitor therapy have been reported.

Reports of HS-associated SCC arising under biologic therapy remain scarce. Here, we present the first reported case of a rapidly progressive, giant SCC developing from long-standing HS during IL-17 inhibitor (bimekizumab) therapy. The malignancy enlarged dramatically within a short period after the initiation of biologic treatment. This case underscores the need for vigilance regarding malignant transformation in HS, particularly in patients receiving newer biologic agents, and prompts discussion on the possible influence of cytokine blockade on tumor behavior.

## Case presentation

**Figure 1:**
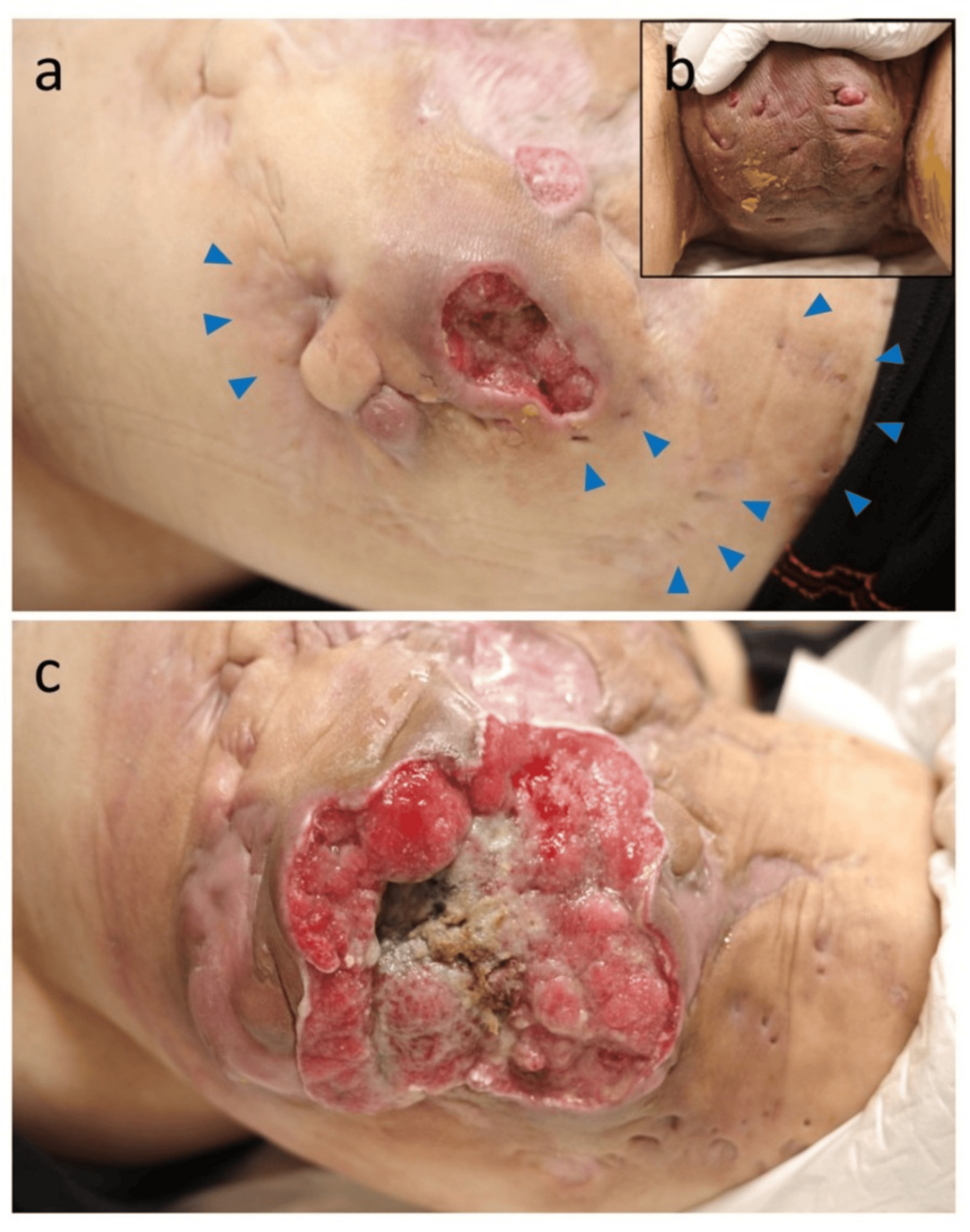
Clinical findings. (a) A chronic 5 cm ulcerative lesion in the left trochanteric region, with multiple surrounding scars (arrowheads) indicative of long-standing hidradenitis suppurativa, persisting for several months before treatment. (b) Severe hidradenitis suppurativa involving the scrotum, showing sinus tracts and nodules, consistent with Hurley stage III disease. (c) Progression of the lesion shown in a: at presentation to our hospital (approximately four months after bimekizumab initiation), the ulcer had enlarged to 15 cm with heaped-up margins and multiple firm subcutaneous nodules surrounding the primary tumor.

A man in his 50s with a 20-year history of HS affecting both buttocks presented with a rapidly enlarging ulcerated mass on the left hip (trochanteric area). His medical history was notable for a T12 spinal cord injury in his 20s, resulting in paraplegia. He had a history of smoking and had not received adalimumab or other biologic therapies prior to bimekizumab. During the past 20 years, he experienced recurrent nodules, abscesses, and purulent discharge in the buttock and perineal regions, and multiple scars and sinus tracts had developed around the trochanteric and gluteal areas, consistent with long-standing HS. Four months before this presentation, he had been started on the IL-17 inhibitor bimekizumab at another clinic to treat a severe HS flare (Hurley stage III disease). At that time, a chronic 5 cm ulcerative lesion with surrounding scars was already present in the left trochanteric area and had persisted for approximately three months (Figure 1a). The patient was being treated at another clinic, and although the precise reasoning is unknown, the lesion was likely judged to be consistent with long-standing HS given the presence of multiple scars and sinus tracts, and therefore, a biopsy was not performed at that stage. In addition, multiple HS-related lesions were evident on the scrotum, demonstrating the extent of severe disease (Figure 1b).

Within two months of beginning bimekizumab, the left trochanteric lesion underwent rapid, exophytic growth, increasing dramatically in size and raising concern for malignant transformation (Figure 1c). A biopsy confirmed SCC, and bimekizumab therapy was promptly discontinued. The patient was then referred to our hospital for definitive management. On examination at presentation (approximately three months after bimekizumab initiation), the ulcerating tumor measured approximately 15 cm in diameter with heaped-up margins and multiple firm subcutaneous nodules surrounding the primary lesion. Contrast-enhanced computed tomography showed bilateral inguinal lymphadenopathy but no evidence of distant metastasis. Lymphoscintigraphy identified a sentinel lymph node in the left inguinal region.

**Figure 2:**
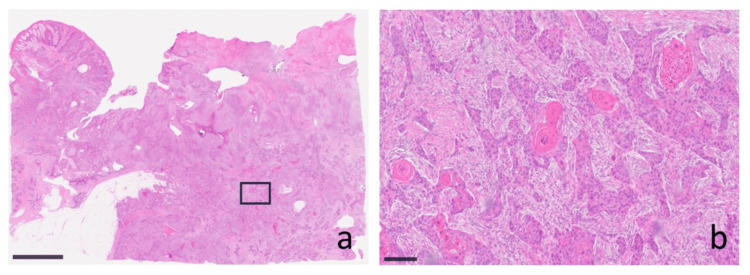
Histopathological findings of the resected tumor. (a) Low-power view showing an exophytic and infiltrative tumor with irregular nests extending into the deep dermis and subcutaneous tissue, accompanied by surrounding fibrosis and chronic inflammation (hematoxylin and eosin staining; scale bar = 5 mm). (b) High-power view demonstrating well-differentiated squamous cell carcinoma with keratin pearls, atypical squamous cells with intercellular bridges, and a desmoplastic stromal reaction (hematoxylin and eosin staining; scale bar = 200 µm).

The patient underwent wide local excision of the left trochanteric tumor with 2 cm lateral margins, including en bloc resection down to the gluteal fascia. A concurrent sentinel lymph node biopsy was performed for the left inguinal basin. Intraoperatively, the mass was found to extend to the level of the fascia but was resected with clear margins. The surgical defect was reconstructed with a split-thickness skin graft. Histopathological examination confirmed an invasive, high-grade SCC (Figure 2a, 2b). All resection margins were free of tumor, and no metastasis was found in the excised inguinal sentinel node. The postoperative course was uneventful. The skin graft achieved complete take by one month after surgery. Adjuvant radiotherapy to the local site was initiated at two months postoperatively. At the three-month follow-up, the patient remained free of local recurrence or distant metastasis, and the surgical site had healed without complications. Functional status was preserved without difficulties in sitting, and no pressure ulcers developed.

## Discussion

HS lesions, particularly in long-standing and severe cases, are often referred to as “immunocompromised districts” due to local immune dysregulation, creating a microenvironment prone to malignant transformation. HS-associated SCC is recognized to have a more aggressive clinical course than conventional cutaneous SCC, with higher rates of regional spread and disease-specific mortality. A recent patient-level meta-analysis of 138 cases showed that nearly half developed regional or distant metastases, with disease-specific mortality approaching 50%, underscoring the high aggressiveness of these tumors even after complete excision with negative margins [2,7].

A recent systematic review identified HS-associated SCC in up to 4.6% of patients with long-standing Hurley stage III disease, further supporting the recognition of chronic HS as a precancerous condition requiring vigilant monitoring [8]. Furthermore, a nationwide Danish cohort study demonstrated a significantly increased overall cancer incidence in patients with HS (standardized incidence ratio: 1.40), with notably higher rates of skin SCC, oropharyngeal cancer, and central nervous system tumors [9].

Most reported cases arise after decades of active HS and often present at an advanced stage in middle age. A systematic review noted a mean age of ~53 years and an average HS duration of 26 years at SCC diagnosis, with a 37% metastasis rate and 40% mortality rate for HS-associated SCC overall [10]. These findings align with our case, which showed rapid progression in a patient with long-standing HS who was receiving IL-17 inhibitor therapy.

The potential role of biologic therapy in accelerating SCC development warrants consideration. TNF-α inhibitors and other biologics can dampen certain immune pathways, potentially reducing tumor immune surveillance. IL-17 signaling has been increasingly recognized as a key contributor to cancer-associated inflammation. It promotes tumor growth through the recruitment of immunosuppressive myeloid cells, angiogenesis, and resistance to apoptosis, although context-dependent anti-tumor effects have also been described [11,12]. Differential IL-17 receptor subunit expression among cancer types may further modulate CD8⁺ T-cell infiltration and immune responsiveness [13].

Importantly, large-scale meta-analyses and population-based studies have shown no overall increase in malignancy risk with IL-17 or IL-23 inhibitors; some even suggest a potential reduction in certain cancer types compared with TNF-α inhibitors or the general population [14]. While causality cannot be established based on a single case, our report highlights the importance of ongoing pharmacovigilance and the accumulation of further cases, especially in patients with chronic HS treated with novel biologic agents.

Recognizing malignant transformation in the context of chronic HS can be challenging, as neoplastic changes may be masked by chronic inflammation and scarring [11]. Clinicians should maintain a high index of suspicion when lesions exhibit rapid growth, refractory ulceration, or induration, particularly in patients receiving biologics. The early biopsy of any suspicious chronic lesion is essential. Once HS-associated SCC is diagnosed, aggressive surgical planning and management are crucial.

## Conclusions

HS-associated SCC is a rare but highly aggressive malignancy that often arises in patients with long-standing, severe disease. Our case highlights the diagnostic challenge of detecting malignant transformation within chronically inflamed and scarred tissue, as well as the potential for rapid progression once SCC develops. Although IL-17 inhibitors such as bimekizumab are generally regarded as carrying a low risk of malignancy, this report represents the first documented case of HS-associated SCC arising during such therapy, raising important questions regarding the potential influence of cytokine blockade on tumor behavior. Clinicians should maintain a high index of suspicion for non-healing, indurated, or rapidly enlarging lesions in patients with HS, particularly those receiving biologic agents, and pursue early biopsy when warranted. Vigilant clinical monitoring and timely surgical intervention remain essential to improving outcomes in this challenging patient population.
